# Remdesivir treatment for patients with moderate to severe COVID-19

**DOI:** 10.55730/1300-0144.5387

**Published:** 2022-04-16

**Authors:** İmran HASANOĞLU, Rahmet GÜNER, İlhami ÇELİK, Fikret KANAT, Ayşe BATIREL, Gülçin TELLİ DİZMAN, Esma EREN, Dilek YILDIZ SEVGİ, İlkay BOZKURT, Kadriye KART YAŞAR, Sevtap ŞENOĞLU, Esra KAZAK, Rıdvan KARAALİ, Aysel ÇELİKBAŞ, Hüsnü PULLUKÇU, Arif Atahan ÇAĞATAY, Serhat ÜNAL, Şebnem ERDİNÇ, Fehmi TABAK, Ahmet GÜL, Emine ALP

**Affiliations:** 1 Department of Infectious Diseases and Clinical Microbiology, Faculty of Medicine, Yildirim Beyazit University, Ankara City Hospital, Ankara, Turkey; 2 Department of Infectious Diseases and Clinical Microbiology, University of Health Sciences, Kayseri City Hospital, Kayseri, Turkey; 3 Department of Pulmonary Diseases, Faculty of Medicine, Selcuk University, Konya, Turkey; 4 Department of Infectious Diseases and Clinical Microbiology, University of Health Sciences, Kartal Dr. Lutfi Kırdar City Hospital, İstanbul, Turkey; 5 Department of Infectious Diseases and Clinical Microbiology, Faculty of Medicine, Hacettepe University, Ankara, Turkey; 6 Department of Infectious Diseases and Clinical Microbiology, University of Health Sciences, İstanbul Şişli Hamidiye Etfal Education and Research Hospital, İstanbul, Turkey; 7 Department of Infectious Diseases and Clinical Microbiology, Faculty of Medicine, Ondokuz Mayıs University, Samsun, Turkey; 8 Department of Infectious Diseases and Clinical Microbiology, University of Health Sciences, Bakırkoy Dr Sadi Konuk Education and Research Hospital, İstanbul, Turkey; 9 Department of Infectious Diseases and Clinical Microbiology, Faculty of Medicine, Uludağ University, Bursa, Turkey; 10 Department of Infectious Diseases and Clinical Microbiology, Cerrahpaşa School of Medicine, İstanbul University, İstanbul, Turkey; 11 Department of Infectious Diseases and Clinical Microbiology, Faculty of Medicine, Hitit University, Çorum, Turkey; 12 Department of Infectious Diseases and Clinical Microbiology, Faculty of Medicine, Ege University, İzmir, Turkey; 13 Department of Infectious Diseases and Clinical Microbiology, Faculty of Medicine, İstanbul University, İstanbul, Turkey; 14 Department of Infectious Diseases and Clinical Microbiology, University of Health Sciences, Ankara Education and Research Hospital, Ankara, Turkey; 15 Department of Rheumatology, Faculty of Medicine, İstanbul University, İstanbul, Turkey; 16 Ministry of Health, Ankara, Turkey

**Keywords:** COVID-19, remdesivir, antiviral, treatment, mortality

## Abstract

**Background/aim:**

Remdesivir, which was first developed for the treatment of Ebola disease but failed to meet expectations, has become hope in the fight against the COVID-19 pandemic. This study aimed to evaluate risk factors for mortality and prognosis of adult moderate/severe COVID-19 patients treated with remdesivir, and safety and tolerability of 5 days of remdesivir treatment.

**Materials and methods:**

This multicenter prospective observational study was conducted in 14 centers in Turkey. Pregnancy or breastfeeding, multiorgan failure, or usage of vasopressors for septic shock, ALT > 5 × the upper limit of the normal range, or eGRF <30 mL/min or dialysis and receiving favipiravir were the exclusion criteria of the study.

**Results:**

Among 500 patients, 494 patients were included in the study. On admission, 392 (79.3%) patients had moderate and 102 (20.6%) patients had severe COVID-19. The 28-day mortality was 10.1%. The median of the scores of the seven-category ordinal scale assessed on days 0, 3, 5, 7 were 4 and 3 on day 14. When the survival status of the patients was evaluated according to the time between the remdesivir start date and the end date of the symptoms, no statistically significant difference was found between the medians of the groups (p = 0.404). In multivariable analysis, age (OR, 1.05; 95%CI, 1.02–1.08; p = 0.003), SpO_2_ level on admission (OR, 3.03; 95%CI, 1.35–6.81; p = 0.007), heart rate (OR, 2.48; 95%CI, 1.01–6.07; p = 0.047), follow-up site at the hospital (clinic/ICU) (OR, 26.4; 95%CI, 11.6–60.17; p < 0.001) were independently associated with increased mortality. Grade 3 adverse event (AE) was observed in 4 (0.8%) patients. None of the patients experienced grade 4 or 5 AEs.

**Conclusion:**

Remdesivir is a safe and well-tolerated drug and older age, low SpO_2_ level on admission, tachycardia, and ICU admission are independently associated with increased mortality among patients with moderate/severe COVID-19 receiving remdesivir treatment.

## 1. Introduction

The second pandemic of the 21st century has directed almost all resources of medicine to treatment and vaccination studies. Although tremendous success has been achieved in terms of vaccines for COVID-19, it is still not possible to say the same for the antiviral treatment. SARS-COV-2 often causes a mild upper respiratory tract infection; nevertheless, it is a complicated disease with a hyperinflammatory response that becomes a challenge to physicians in many respects. Although there are treatments that have been shown to be effective in the host inflammatory response phase, antiviral treatments are particularly important in the early viral response phase of the disease to be able to block viral replication.

Remdesivir, a nucleotide analogue of adenosine 5-monophosphate, is a broad-spectrum antiviral against RNA viruses like severe acute respiratory syndrome coronavirus 1 (SARS-CoV-1), Middle East respiratory syndrome virus (MERS-CoV), and Ebola virus [1]. It is a potent inhibitor of viral RNA-dependent RNA polymerase, which causes the termination of RNA transcription after it is taken up by the virus into RNA strands. Early in the COVID-19 pandemic, researchers reported that remdesivir has an in vitro activity against SARS-CoV-2 [2], and then remdesivir became one of the arms of randomized controlled studies like Solidarity and Adaptive COVID-19 Treatment Trial (ACTT-1). Early reports of the ACTT-1 trial in April 2020 showed that remdesivir shortens the duration of complaints (11 vs 15 days) in COVID-19 cases with pneumonia. The final results of the ACTT-1 study concluded that remdesivir was superior to placebo in shortening the time to recovery of adults who were hospitalized due to COVID-19 and carried symptoms of lower respiratory tract infection [3], which resulted in its approval by the Food and Drug Administration (FDA) as the only antiviral drug for the treatment of hospitalized COVID-19 patients.

This study aimed to evaluate: 1-the prognosis of adult patients who have been diagnosed with moderate to severe COVID-19 and treated with remdesivir, 2-risk factors for mortality among moderate to severe COVID-19 patients treated with remdesivir, 3-safety and tolerability of 5 days of remdesivir treatment.

## 2. Materials and methods

### 2.1. Study design

This multicenter prospective observational study was conducted in 14 referral centers for COVID-19 in Turkey in compliance with the Declaration of Helsinki and Good Clinical Practice. The study was approved by the Ministry of Health, the ethics board of Ankara City Hospital (No: E1-20-811), and the National Regulatory Agency; and it was conducted between June 30, 2020, and March 05, 2021. A central database was implemented for data collection, and an independent data and safety monitoring board assessed the integrity of the data. All patients provided written informed consent. In the cases where a patient was unable to provide consent, it was obtained from the patient’s legal representative.

### 2.2. Patients and treatment schedule

Moderate and severe COVID-19 patients were identified according to the WHO guideline^1^

#### Inclusion criteria

Patients meeting all of the following criteria were included in the study:

- Diagnosis of COVID-19 with PCR and/or other accepted serological methods accompanied by appropriate clinical complaints and/or findings consistent with COVID-19 in chest CT,
- One of the following in addition to fever or signs of respiratory infection:
  ○ Respiratory rate >30/min,
  ○ Severe respiratory distress (dyspnea, use of extra respiratory muscles),
  ○ More than 50% involvement of lung parenchyma in CT,
  ○ SpO_2_ ≤ 94% in room air,
- ≥18 years old.

#### Exclusion criteria

Patients meeting any of the following criteria were excluded from the study:

- Pregnant or breastfeeding mothers,
- Patients aged <18,
- Multiorgan failure,
- Using vasopressors for septic shock,
- ALT > 5 × the upper limit of the normal range,
- eGRF < 30 mL/min or dialysis or continuous veno-venous hemofiltration,
- Using favipiravir.

Patients were intravenously administered remdesivir as a 200-mg loading dose on the first day and 100-mg maintenance doses on days 2 through 5. Demographic features (e.g., age, sex), symptoms with their onset times, comorbidities, other medications, vital signs, and other physical examination results were recorded.

The clinical assessment was recorded on day 0, day 3, day 5, day 7, and day 14 if the patient was still hospitalized. Patients’ clinical status was categorized as one of a seven-category ordinal scale [4]: 1: not hospitalized, resumed normal activities; 2: not hospitalized, unable to resume normal activities; 3: hospitalized, does not require supplemental oxygen; 4: hospitalized, requires supplemental oxygen; 5: hospitalized, requires nasal high-flow oxygen therapy, noninvasive mechanical ventilation, or both; 6: hospitalized, requires ECMO, invasive mechanical ventilation, or both; and 7: death.

Polymerase chain reaction (PCR) test was performed on day 0, day 3, day 5, day 7, and on day 14 if the patient was still hospitalized.

All serious adverse events and adverse events of grade 3 or 4 that represented an increase in severity when compared to day 1 were recorded.

### 2.3. Study outcomes

The primary outcome of this study was to evaluate the clinical status of the patients on day 28, who were diagnosed with moderate to severe COVID-19 and were treated with remdesivir. The secondary outcomes were independent mortality risk factors among moderate to severe COVID-19 patients who have been treated with remdesivir and evaluation of safety, in particular adverse events that lead to premature treatment discontinuation of remdesivir. The number and the characteristics of adverse events (AE) and serious adverse events (SAE) were monitored. Adverse events classification version 5.0 of the National Cancer Institute Common Terminology Criteria for Adverse Events was used.

### 2.4. Statistical analysis

Statistical analyses were performed using IBM SPSS V25 (SPSS Statistics for Windows, v25.0, IBM Corp, Armonk, NY). Descriptive features of patients are presented as number, percentage, mean ± standard deviation, and median (minimum–maximum) values. Before the statistical analyses, the conformity of the continuous variables to the normal distribution was checked according to the groups, and the analyses were carried out with parametric tests in cases where the conformity to the normal distribution was achieved, and with nonparametric tests when the conformity to the normal distribution was not achieved. Pearson’s chi-squared test or Fisher’s exact test was used to evaluate categorical data. Independent sample t-test/Mann–Whitney *U* test was used for mean/median comparisons of two independent groups. Laboratory results and symptoms of the volunteers were recorded repeatedly. Nonparametric tests were not used for repeated measurements so that the findings would not be misleading due to missing observations, and only comparisons of the first measurements according to the survival status were reported.

Logistic regression analysis (backward-LR method) was used for the multivariable assessment of the relationship between mortality and the various risk factors. The odds ratio (OR) and the 95% confidence interval (CI) were also calculated. A p-value of less than 0.05 was considered statistically significant.

## 3. Results

Demographic and clinical characteristics of the patients are given in Table 1. Among 500 patients who consented to the trial, only 6 patients were excluded from the analysis since they used remdesivir for a shorter period than planned (2 patients 1 day, 4 patients 2 days). Among the remaining patients, 305 (61.7%) were male and 189 were female. On admission, 392 (79.3%) patients had moderate and 102 (20.6%) patients had severe COVID-19. At the time of admission, 452 (91.3%) patients were hospitalized in the clinic and 43 (8.7%) were admitted to the intensive care unit (ICU). Among 452 patients, 85 (18.8%) patients were transferred to the ICU during the follow-up. The 28-day mortality was 10.1% (50 patients) (2.5% for patients followed in the clinic, 47.1% for the patients followed in the ICU). Four patients were still hospitalized on day 28.

SARS-CoV-2 PCR test was positive in 253 (72.3%) patients on admission, 134 patients (42.5%) on the 3rd-day visit, 90 patients (30.5%) on the 5th-day visit, 45 patients (23.0%) on the 7th-day visit, and 8 patients (19.5%) on the 14th day visits. The mortality rate was found to be high in patients with positive PCR test results on admission (p = 0.001).

When the patients were evaluated in terms of comorbidities, hypertension (41.3%) was the most frequent disease, which was followed by diabetes mellitus (29.4%) and chronic cardiac disease (CCD) (21.9%). The median time from illness onset to admission was 4 days. On chest CT, 12 (3.0%) patients had unilateral, 372 (93.5%) had bilateral ground-glass opacity. In addition to remdesivir, 140 (28.1%) patients received dexamethasone, 117 (23.4%) patients received methylprednisolone, 5 (1%) patients received immune plasma, and 5 (1%) patients received tocilizumab.

The median of the scores of the seven-category ordinal scale assessed on days 0, 3, 5, 7 were 4 and 3 on day 14. The median time to symptom resolution from the first administration of remdesivir was 5 [3–9] days and the median time to negative PCR result was 6 [4–9] days.

There was no statistically significant difference between male and female patients in terms of age and 28-day mortality (p=0.173 and 0.930, respectively). The mortality rate in patients with diabetes, renal failure, and chronic lung disease was high (p=0.017, p < 0.001, and p = 0.020, respectively).

When the survival status of the patients was evaluated according to the time between the remdesivir start date and the end date of the symptoms, no statistically significant difference was found between the medians of the groups (p = 0.404). No statistically significant correlation was found between the time to PCR negativity and mortality.

Univariate analysis revealed 11 risk factors to be statistically significant predictors of mortality. The logistic regression model has included 11 regressors that were age, sex, renal failure, diabetes, chronic cardiac disease, chronic lung disease, dyspnea on admission, follow-up site (clinic or ICU), heart rate (>100/min), presence of any comorbidity, and SpO_2_ (<90%) on admission. The model with the backward LR method was completed in 8 steps. Result of the last step is presented in Table 2. The Hosmer–Lemeshow test results showed that the last model adequately fits the data with *X*^2^ = 4.412 and p = 0.818 values. The correct classification rate (Accuracy) of the logistic regression model was found to be 92.1% (Table 3). In multivariable analysis, the following variables were independently associated with increased mortality: age (OR, 1.05; 95% CI, 1.02–1.08; p = 0.003), SpO_2_ level on admission (OR, 3.03; 95% CI, 1.35–6.81; p = 0.007), heart rate (OR, 2.48; 95% CI, 1.01–6.07; p = 0.047), follow-up site at the hospital (clinic or ICU) (OR, 26.4; 95% CI, 11.6–60.17; p < 0.001) (Table 2).

A total of 12 drug-related AEs were recorded. Grade 3 AE was observed in 4 (0.8%) patients. None of the patients experienced grade 4 or 5 AEs. All the observed AEs were transaminase elevation. None of the AEs caused remdesivir discontinuation.

## 4. Discussion

Remdesivir, which was first developed for the treatment of Ebola disease but failed to meet expectations, has become hope in the fight against the COVID-19 pandemic. Considering that the preliminary results of its first study were announced with the presence of the President of the United States, remdesivir has been one of the few particularly popular drugs in the history of medicine. Although hopes that remdesivir could be a game-changer in the treatment of COVID-19 were more widespread at the beginning of the pandemic, this notion weakened over time. Now, recommendations regarding the use of remdesivir for the treatment of COVID-19 vary. In October 2020, FDA approved remdesivir use in hospitalized COVID-19 patients ≥ 12 years old and weighing ≥ 40 kg, and Veklury became the first drug that received FDA approval for the treatment of COVID-19. NIH and Infectious Diseases Society of America recommend remdesivir for the treatment of hospitalized patients who require supplemental oxygen^2^,^3^. Opposingly, the WHO recommends against the use of remdesivir^4^ . WHO bases this recommendation on the results of its open-label adaptive Solidarity trial, which is the largest randomized controlled study of remdesivir with 5472 patients from 405 hospitals in 30 countries [5]. Solidarity revealed that 10 days of remdesivir treatment did not reduce the length of stay in the hospital, initiation of ventilation, and mortality compared to the standard of care. Although a huge number of patients were included in Solidarity, it did not affect the decisions of the FDA, EMA, and many other scientific societies recommending the use of remdesivir. Solidarity’s main shortcoming was the lack of categorization of patients in terms of how they received oxygen (i.e. low or high flow). Instead, patients were classified according to whether they did or did not receive oxygen therapy or if they required mechanical ventilation.

Results of the first randomized double-blind placebo-controlled study of remdesivir were reported by Wang et al. from China. Although the study could not reach the planned sample size due to the early control of the pandemic in China, they found that remdesivir has no statistically significant effect on mortality or clinical improvement compared with standard of care [6]. This study included less severe patients when compared to ACTT-1 which advocated the use of remdesivir. Contrary to intuition, this difference did not offer an advantage for remdesivir in the study of Wang et al. [6,7]. Recently published Discovery study, which is a phase 3 multicenter RCT of remdesivir, concluded that remdesivir did not improve the clinical status of the hospitalized COVID-19 patients neither on day 15 nor 29 and did not reduce mortality and SARS-CoV-2 viral load [8]. ACTT-1 trial, which was conducted by NIH and enrolled 1062 patients (15% mild/moderate disease, 85% severe disease) from 60 hospitals, reported that remdesivir has shortened the recovery time (10 days vs 15 days for placebo). The mortality rate was found to be 11.4% in the remdesivir group and 15.2% in the placebo group on day 29 [3]. The mortality rate in our study was 10.1% on day 28, 14% in the study by Wang et al., and 12.5% in the Solidarity trial [5,6]. It is difficult to compare the main outcome of mortality results since the study designs and study populations were quite different. We included moderate and severe COVID-19 patients, and 62.9% of the patients had at least 1 comorbidity and 61.4% of the patients were male. This heterogeneity caused inconsistent data regarding the efficacy of remdesivir. There are as many metaanalyses as clinical studies of remdesivir in the literature, but these metaanalyses should be interpreted very carefully as well [9–14]. Various randomized controlled studies do not offer uniform findings and have inconsistent results in terms of clinical improvement. In addition, the inability to collaborate and share high-quality data due to the increased workload of physicians in the pandemic was also effective. The Living Project, which is a metaanalysis of 2 trials evaluating the treatments of COVID-19, concluded that there is evidence of a positive effect of remdesivir on serious adverse events when compared to the placebo group, but no difference in mortality or nonserious adverse events [15]. Another living systematic review and metaanalysis which includes 7767 patients from 5 randomized controlled studies worldwide reported that remdesivir probably provides little or no difference in mortality, a slight reduction in the need for ventilation while possibly improving the rate of recovery and serious adverse events. For patients not requiring mechanical ventilation, a 5-day course may be preferred instead of the 10-day course for greater benefits, fewer harms, and lower costs [9].

Although the antiviral efficacy of remdesivir had been demonstrated by preclinical studies, not all clinical studies supported these data. In our study, the SARS-CoV-2 PCR test was positive in 253 (72.3%) patients on admission and 8 patients (19.5%) on the 14th-day visits. In a study from Italy, in all 21 patients, RT-PCR was found to be negative on a median of 12 days after the start of remdesivir treatment [16]. Wang et al. reported that remdesivir did not reduce viral load and detectability of SARS-CoV-2 RNA [6]. In our study, a numerically lower detectability of SARS-CoV-2 RNA on day 14th was observed, and it may reflect the natural course of the disease as well.

While the second year of the pandemic was about to end, risk factors associated with mortality in COVID-19 were revealed with thousands of articles published in the literature. However, there are not many studies evaluating these risk factors in patients receiving remdesivir. A metaanalysis evaluating indicators of severe COVID-19 among 69,762 patients from 88 articles, found that older age, dyspnea, and lower SpO_2_ (<89%) on admission, cardiovascular disease, cerebrovascular disease, chronic lung disease, chronic kidney disease, diabetes, hypertension, cancer, and smoking are risk factors for mortality [17]. Another meta-analysis revealed similar risk factors regarding mortality among 423,117 patients from 42 studies and reported the pooled prevalence of mortality among hospitalized COVID-19 patients as 17.62% [18]. In a study from Turkey, which evaluates different treatment regimens including remdesivir (n: 17), researchers reported that the most significant factor for increased mortality is ICU admission [19]. In our study population of moderate/severe COVID-19 patients treated with remdesivir; male sex, renal failure, diabetes, chronic cardiac disease, chronic lung disease, dyspnea on admission, and presence of any comorbidity were found as risk factors for mortality only in univariate analysis. However, in multivariable analysis, age (OR, 1.05; 95% CI, 1.02–1.08; p = 0.003), SpO_2_ level on admission (OR, 3.03; 95% CI, 1.35–6.81; p = 0.007), heart rate (OR, 2.48; 95% CI, 1.01–6.07; p = 0.047), follow-up site at the hospital (clinic or ICU) (OR, 26.4; 95% CI, 11.6–60.17; p < 0.001) were independently associated with increased mortality.

This study also evaluated the safety and tolerability of remdesivir. A total of 12 drug-related AEs were recorded. Grade 3 AE was observed in 4 (0.8%) patients. None of the patients experienced grade 4 or 5 AEs. All the observed AEs were transaminase elevation. According to the product information, nausea and elevation of liver enzymes are the most observed adverse reactions^5^ . None of the AEs caused remdesivir discontinuation. Our study demonstrated that remdesivir is safe and well-tolerated, in accordance with the current literature [20,21]. Drug-related adverse reactions are observed more commonly in 10 days of remdesivir treatment. Antinori et al. reported that elevation of liver enzymes is observed in 42.8% of the patients and one-third of the patients enrolled were unable to complete the 10-day remdesivir treatment due to the presence of AEs [16]. Grein et al. reported similar rates of AEs with a 10-day course of remdesivir [7]. In a randomized, open-label, phase 3 study of remdesivir, 30% of the 200 patients enrolled in the 5-day group experienced no AE of grade 3 or higher [22]. In general, the most observed adverse events were nausea, acute respiratory failure, elevated ALT, and constipation. The ratio of patients who had to discontinue treatment due to adverse events was 4% in the 5-day group and 10% in the 10-day group. However, this comparison is questionable owing to the fact that some studies categorize acute respiratory failure as an adverse event, whereas some do not. Moreover, since COVID-19 also causes acute respiratory failure, detecting a drug-induced acute respiratory failure is subjective. Furthermore, the elevation of liver enzymes, the most common drug-related adverse event, can also be associated with the COVID-19. Therefore, it is very important to interpret the results related to adverse events carefully.

This study has several limitations. The first and the most important one is that the study does not include a placebo or an alternative treatment arm. Therefore, a comparison of remdesivir treatment with the standard of care could not be performed. Due to this limitation, the possibility that patients who clinically improved after remdesivir treatment could have improved even without any treatment cannot be ruled out. Secondly, we included only moderate to severe COVID-19 patients; hence, the results cannot be generalized for all patient groups. Thirdly, the trend of the SARS-CoV-2 viral load under remdesivir treatment was not evaluated. Fourthly, the study was conducted in 14 centers from different cities across Turkey. Since cut-off values for laboratory parameters were different across centers, some laboratory parameters that may have turned out to be outcome predictors were not recorded.

In conclusion, since the most important stage of the pathogenesis is viral replication, there is no doubt that antivirals fit in the center of the puzzle of COVID-19 treatment. Remdesivir is the first drug that received FDA approval for treatment of COVID-19, but different societies have diverse recommendations regarding the use of remdesivir. According to this study, remdesivir is a safe and well-tolerated drug, and older age, low SpO_2_ level on admission, tachycardia, and ICU admission are independently associated with increased mortality among patients with moderate/severe COVID-19 receiving remdesivir treatment.

## Figures and Tables

**Table 1:** Baseline characteristics of the patients.

	All patients	Died (n, %)	Survived (n, %)	p-value
Demographic characteristics				
Age, years (median, min–max)	62.0 [21.0–97.0]	71.0 [26.0–92]	61.0 [21.0–97.0]	U = 7010.0 p < 0.001
Age groups				
18–30 n (%)	15 (100)	1 (6.7)	14 (93.3)	*X^2^* = 9.396 p = 0.024
31–45 n (%)	54 (100)	3 (5.6)	51 (94.4)	
46–65 n (%)	225 (100)	16 (7.1)	209 (92.9)	
≥66 n (%)	196 (100)	30 (15.3)	166 (84.7)	
Sex				
Male n (%)	301 (100)	31 (10.3)	270 (89.7)	*X^2^* = 0.008 p = 0.930
Female n (%)	189 (100)	19 (10.1)	170 (89.9)	
Preexisting conditions				
Any comorbidity	314 (100)	41 (13.1)	273 (86.9)	*X^2^* = 8.165 p = 0.004
Hypertension	204	25 (%12.3)	179 (%87.7)	p = 0.205
Diabetes	144	22 (%15.3)	122 (%84.7)	p = 0.017
Chronic cardiac disease	108	19 (%17.6)	89 (%82.4)	p = 0.004
Chronic pulmonary disease	73	13 (%17.8)	60 (%82.2)	p = 0.020
Renal failure	16	6 (%37.5)	10 (%62.5)	p = 0.001
Malignancy	20	4 (%20)	16 (%80)	p = 0.139
Clinical features				
Fever	160 (100)	18 (11.2)	142 (88.8)	p = 0.594
Cough	283 (100)	28 (9.9)	255 (90.1)	p = 0.791
Dyspnea	97 (100)	17 (17.5)	80 (82.5)	p = 0.008
Sore throat	35 (100)	3 (8.6)	32 (91.4)	p = 0.741
Headache	52 (100)	5 (9.6)	47 (90.4)	p = 0.882
Myalgia	100 (100)	12 (12.0)	88 (88.0)	p = 0.506
Diarrhea	43 (100)	2 (4.7)	41 (95.3)	p = 0.208
Nausea/vomiting	56 (100)	5 (8.9)	51 (91.1)	p = 0.738
Heart rate > 100/min	72 (100)	16 (22.2)	56 (77.8)	*X^2^* = 15.03 p < 0.001

**Table 2:** Logistic regression model for mortality prediction.

	B	S.E.	Wald	df	p-value	OR	95% CI for OR	
							Lower	Upper
Age	0.046	0.016	8.536	1	0.003	1.047	1.015	1.080
SpO_2_ level on admission	1.107	0.414	7.167	1	0.007	3.026	1.345	6.807
Hearth rate	0.908	0.457	3.958	1	0.047	2.481	1.014	6.071
Follow up site at the hospital	3.273	0.420	60.629	1	<0.001	26.397	11.581	60.169
Constant	–7.185	1.163	38.178	1	<0.001	0.001		

a Variable(s) entered on step 1: Sex _(male/female)_, age _(continious)_, any comorbidity _(no/yes)_, diabetes mellitus _(no/yes)_, chronic cardiac disease _(no/yes)_, chronic lung disease (asthma, /COPD etc.) _(no/yes),_ dispnea _(no/yes)_, SpO_2_ level on admission to the hospital _(intermediate level/severe level)_, heart rate _(≤100/100<),_ follow-up site at the hospital _(clinic/ICU)_, renal failure_(no/yes)_, p < 0.05 significance level

**Table 3:** Classification table of the logistic regression model.

Observed			Predicted		
			Survival		Percentage correct
			Survived	Died	
Last step	Survival	Survived	423	12	97.2
		Died	26	19	42.2
	Overall percentage				92.1

## Footnotes

1 Clinical management of COVID-19. Website https://www.who.int/publications-detail-redirect/clinical-management-of-covid-19 (accessed 25 December 2021).

2 IDSA Guidelines on the Treatment and Management of Patients with COVID-19. Website https://www.idsociety.org/practice-guideline/covid-19-guideline-treatment-and-management (accessed 4 October 2021)

3 Therapeutic Management COVID-19 Treatment Guideline. Website https://www.covid19treatmentguidelines.nih.gov/management/clinical-management/hospitalized-adults--therapeutic-management/ (accessed 4 October 2021).

4 COVID-19 Clinical management: living guidance. Website https://www.who.int/publications/i/item/WHO-2019-nCoV-clinical-2021-2 (accessed 23 December 2021).

5 Veklury Prescribing Information. Website https://www.gilead.com/-/media/files/pdfs/medicines/covid-19/veklury/veklury_pi.pdf (Accessed 12 December 2021)
